# Position-resolved charge collection of silicon carbide detectors with an epitaxially-grown graphene layer

**DOI:** 10.1038/s41598-024-60535-3

**Published:** 2024-05-06

**Authors:** Ivan Lopez Paz, Philippe Godignon, Neil Moffat, Giulio Pellegrini, Joan Marc Rafí, Gemma Rius

**Affiliations:** Instituto de Microelectronica de Barcelona, IMB-CNM-CSIC, 08193 Cerdanyola del Valles, Barcelona, Spain

**Keywords:** Sensors and biosensors, Characterization and analytical techniques

## Abstract

Silicon carbide (SiC) has outstanding physical properties therefore, diodes based on SiC are being considered for many radiation detection applications such as particle accelerator experiments and medical dosimetry. Moreover, by reducing the metal on the surface of the diode there is the potential to enhance its performance in some fields where the presence of metal is detrimental. To this end, SiC detectors with an epitaxially-grown graphene layer (EG), that substitutes the metallic contact, in the sensitive region were produced at IMB-CNM, profiting from the conductivity of the mono-atomic layer material. To isolate the effect of the graphene on the charge collection, samples without graphene were produced in parallel. In this paper, the effect of EG on Silicon Carbide p-in-n radiation detectors is studied in terms of charge collection with a radioactive source and by means of the transient current technique (TCT), which allows for position-dependent signal formation analysis. As a result of the former, we show the capability of the EG-SiC sensor for charge collection after signal integration, to a resolution close to that of a sensor fully metallised. Moreover, from the TCT studies, we observe uniform charge collection across the active region, as well as an up-to $\sim$40% transient amplitude damping which, compared with the $\sim$90% on the sample containing no metallic contact, proves that the presence of graphene benefits the performance of the device and that the technology is viable for radiation detection as an alternative to metal.

## Introduction

Silicon carbide is growing in interest as a solid-state radiation detector material alternative to silicon^1^ due to the availability of high quality 6–8 in. wafers, due to recent advances in power devices. With a higher displacement energy (22–35 eV) and electron saturation velocity (2.2 $\times$ 10^17^ cm/s)^2^ than Silicon (13–20 eV and 0.86 $\times$ 10^17^ cm/s respectively), SiC has potential use in many applications where fast response and radiation hardness are key challenges. In addition, a high thermal conductivity [370 W/(m K)] alleviates the need for cooling, and allows for high temperature applications.

Typical SiC radiation detectors consist of a pn-junction, produced by the implantation of dopant ions, which is contacted by a metallic structure from which the resulting signal formed by ionising impinging radiation is collected^3^. However, in some applications the presence of metal is undesirable, such as in radiotherapy dosimeters, introducing X-ray scattering; or low energy ion detection, where metal structures can absorb a fraction of the charged particle energy. A possible solution to reduce this effect is the replacement of the metallic contacts in the active region with a non-metallic electrically conductive layer, such as graphene.

Graphene is an atomic carbon layer, which has interesting properties such as high electron mobility at room temperature and high thermal conductivity^4^. There has been an effort to pursue the implementation of graphene in Silicon devices^5^. In terms of radiation sensors, graphene on Silicon has been used e.g. for deep ultraviolet photodetection^6,7^, reducing the absorption probability of the impinging light with respect to its metallised alternative. Similar technologies have been implemented thanks to the development of processes for the formation of graphene layers in Silicon Carbide by epitaxial growth^8^. For instance, reference^9^ reports the performance of a SiC device with epitaxial graphene in Schottky mode as a UV photodiode.

In this paper we investigate the effect of an epitaxially-grown graphene layer replacing the metallic contact over the active region in SiC diodes as radiation detectors. In particular, detector prototypes showcasing this technology are characterised in terms of charge collection and signal characteristics both with a radioactive source and by means of a UV pulsed laser to obtain position-resolved information. This device is compared against two reference samples: a diode without any contact layer over the active region—except a bias metallic ring—and a second diode with a metallic layer.

## Sensor design and materials

**Figure 1:**
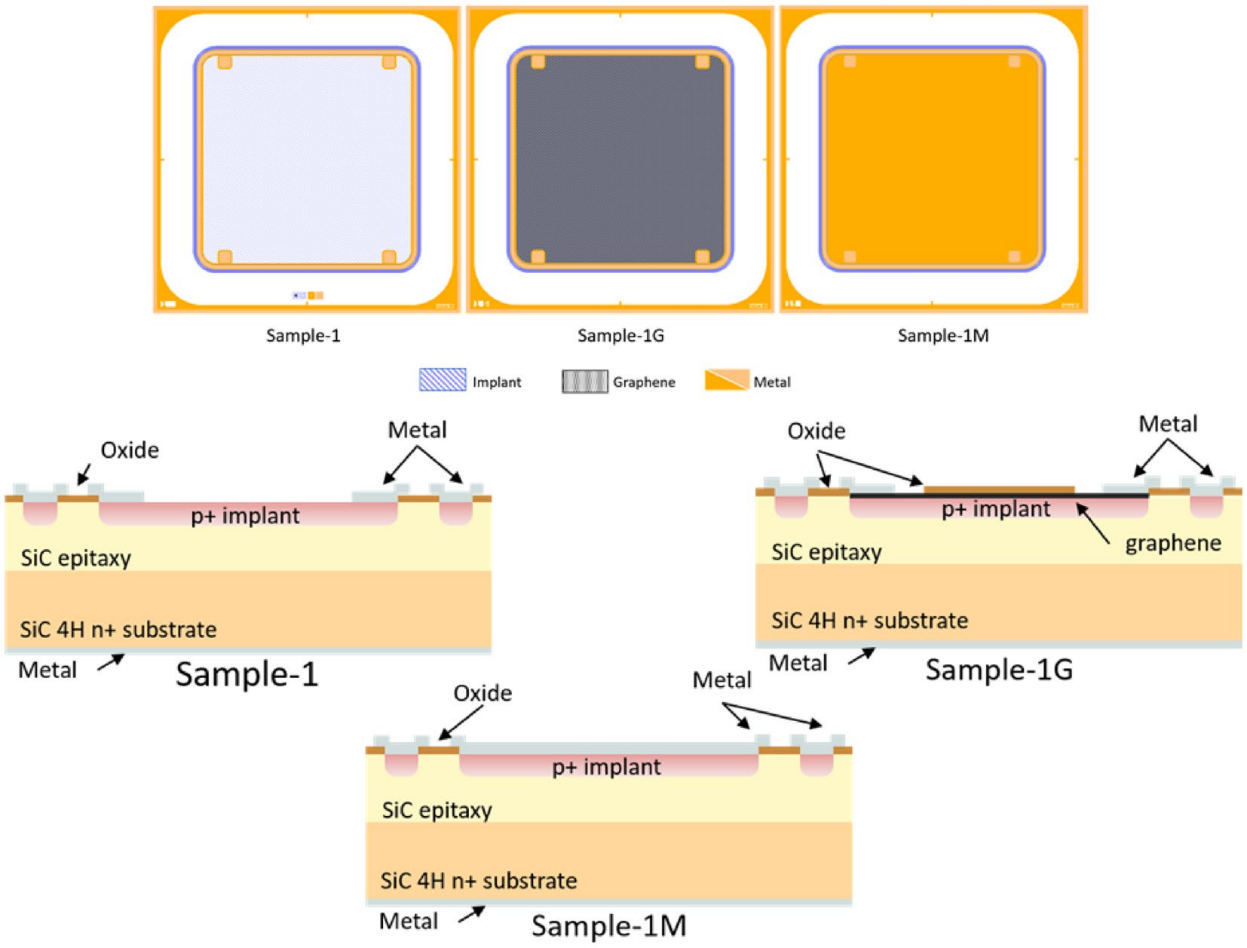
Schematic showing the front side layout (top) and cross-section layer disposition (bottom) in samples without neither graphene nor metal over the implant, with graphene and with fully metallised active region. Note that implant area and graphene/metal overlap in Samples-1G and -1M. Wirebonding is performed onto the metal layer.

A quarter of a 4-in. silicon carbide wafer with a 50 $\upmu$m high resistivity n-type epitaxy layer was used to produce the diodes. The 4H-SiC wafer was purchased from II–VI^10^, where the epitaxial layer was grown by CVD over an N-doped substrate (>10^18^ cm^-3^). Aluminium (Al) was implanted via ion beam onto the SiC surface as a p-type electrode to achieve the pn-junction. Among the structures fabricated therein, three 3 $\times$ 3 mm^2^ square diodes were selected for the studies presented in this paper: (a) a diode with metal contacting the implant over its active region—referred to as “Sample-1M” hereafter—, (b) a sample with a 12 $\upmu$m wide ring around the edge of the implant to contact the pn-junction—“Sample-1”—and, finally, (c) a device featuring a graphene layer between the Al implant and the 12 $\upmu$m wide metal ring—“Sample-1G” (see Fig. 1, top). As they form part of the same wafer, regardless on their layout , all samples have been exposed to the same thermal processes, and thus, this selection allows to isolate and study the effect of the graphene layer in the particle detection process.

In structures of the type of Sample-1G, graphene layers were grown from the Al-implant side. This is realised by sublimating the silicon atoms at high temperatures (>1500 ^∘^C), while the carbon atoms left in the sample re-arrange to produce honeycomb-structured layers^8,11^.

This process is followed by an oxide layer deposition of Al_2_O_3_ and SiO_2_ of 60 nm and 300 nm respectively which are etched to allow for interconnection between metal and implant. The active area surface containing graphene was not removed to prevent over-etching that could damage the graphene layer. Afterwards, a metallic stack of Ti/Pt/Au (20 nm/100 nm/100 nm) is deposited by sputtering over the implant and the graphene layers (when applicable) allowing connection via wirebonding. As aforementioned, in this step for diodes of type Sample-1 and -1G, the front metallisation consists a 12 $\upmu$m-wide ring around the implant (see Fig. 1, top), produced using the lift off process. Similarly, the back side of the wafer was fully metallised by sputtering with a Ti/Ni/Ti/Ni/Au metal stack to contact the n^+^ side of the diodes.

The schematic in Fig. 1 (bottom) showcases the difference in the cross-sectional design between each of the devices from the same wafer, as a result of the fabrication process previously described.

After fabrication, the selected diodes were diced from the wafer and glued onto a PCB with conductive glue in order to allow electric connectivity with the back-side of the sample, and afterwards the top side was wirebonded to the PCB.

**Figure 2:**
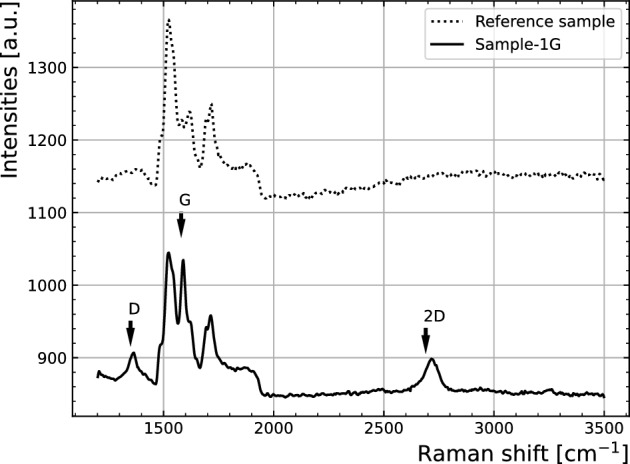
Raman spectra of a reference diode without graphene and metal (dashed line) and Sample-1G. The characteristic peaks of graphene are indicated with arrows.

The verification of graphene presence in samples is performed by Raman spectroscopy. Sample-1G was measured in a Horiba XploRA-plus Raman spectrometer^12^, fitted with a laser of wavelength 532 nm, applying 25% filter to the emitted laser power and a 1200 gratings/mm. For reference, the spectrum obtained from an equivalent substrate (similarly doped Silicon Carbide sample), but with no graphene on its surface is also taken. Both spectra are presented in Fig. 2. Characteristic Raman peaks for (single layer) graphene films are expected to be found at $\sim$1580 cm^-1^ (G band), $\sim$2690 cm^-1^ (2D band) and $\sim$1350 cm^-1^ (D band) when a 532 nm excitation laser is used^13^. The Sample-1G spectrum is an exemplary data, representative of the area coverage of graphene, and allows unambiguously to identify and analyse 2D and G graphene bands, as well as the D peak. Having a reference SiC spectrum, which overlaps to the graphene fingerprint spectrum, helps assessing quantifications of the graphene peaks, as it can be subtracted as baseline counts to perform graphene peaks fittings, as follows.

The ratio of G/2D peak intensities and FWHD of 2D clearly indicate that in average it consists of more than one graphene layer, estimated as 2 or 3 in average. The results also show that G and 2D bands are centred at about 1588 cm^-1^ and 2715 cm^-1^ respectively, thus accounting for some strain, which is often found in epitaxial graphene grown upon the Si face of SiC wafers and is affected by the presence of the buffer layer. The presence of some D band, often referred as defects peak, accounts for differences with respect to ideal or pristine graphene film, in terms of e.g. single crystal grain size, doping or device processing-related structural damage or contamination.

## Methods

To study the prototypes described in the previous section, the transient current and charge collection are studied by irradiating the devices under a radioactive source^14,15^, and by means of the transient current technique (TCT)^16,17^.

### Radioactive source analysis

In order to initially observe charge collection, a triple alpha radioactive source—^239^Pu/^241^Am/^244^Cm— is used. The resulting alpha particles have a kinetic energy of 5.245 MeV, 5.486 MeV and 5.902 MeV respectively. The radioactive source was placed at 8 mm from the devices in air, thus the particles lose a fraction of that energy before reaching the detectors, with an energy down to 4.528 MeV, 4.793 MeV and 5.250 MeV respectively^18^.

Sample-1G has an oxide layer over the graphene, as described in in the previous section. The estimated energy absorption in the oxide is about 60–70 keV. On the other hand, although no oxide layer is present in the active region of Samples-1 and -1M, the later features a thin three-metal stack, which absorb about 100 keV. In all cases, all the energy is deposited at a depth of 12–17 $\upmu$m^18^, well within the active volume of the samples.

The detectors under test were connected to a Bias-T to decouple the leakage current from the signal produced by the alpha particles, the later being amplified with a Cividec C2-TCT current-sensitive amplifier with a 40 db amplification and 10 kHz to 2 GHz bandwidth^19^. The amplified signal is collected by a 5 GSa/s and 700 MHz bandwidth DRS4 Evaluation board^20^, set with a threshold of 50 mV in order to remove noise contributions.

After baseline correction, the charge collected is determined by integrating the waveform resulting from the readout chain.

### Transient current technique

**Figure 3:**
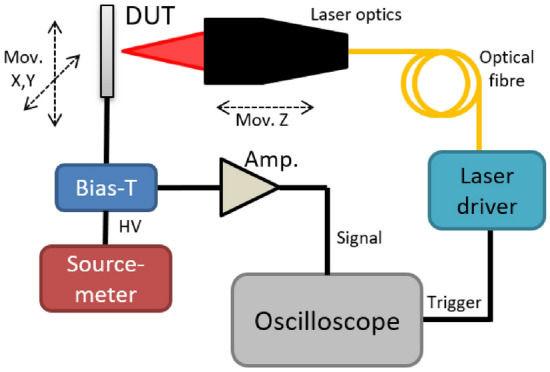
Transient current technique set-up schematic.

Position dependent charge collection is studied via the transient current technique^16,17^. A diagram of the set-up is shown in Fig. 3. The detector under test is illuminated with a UV ($\lambda =$ 369 nm) pulsed laser of $\sim$100 ps width which generates charge carriers inside the SiC. Such wavelength corresponds to a penetration depth of 230 $\upmu$m^21^ in this material. The laser power has been measured to be 26±5 pW with a 1 kHz repetition rate, thus a pulse energy of 26±5 fJ was focused on the sample surface.

This results in a transient signal, which is decoupled from the DC leakage current with a Bias-T, and amplified with a AM-02A current-sensitive amplifier with an amplification of 53 dB and a bandwidth of 0.01–3000 MHz from particulars^22^. Signals after the amplification step, are measured using a 8 GHz, 40 GSa/s oscilloscope (Agilent infiniium DSA90804A), which is triggered by the TTL-level signal trigger output from the laser driver (Pilas DX). Each waveform read out from the oscilloscope is the result of the averaging of 1000 pulses for every position and voltage. To focus the laser beam onto the device under test, the PCB containing the sample is mounted on a two-axis movable stage, perpendicular to the beam direction, while the optical system is installed in a one axis stage. All elements in the set-up are controlled with a custom built Python software.

It is important to note that, due to the presence of metal over all the active region, it was not possible to test Sample-1M under these conditions.

**Figure 4:**
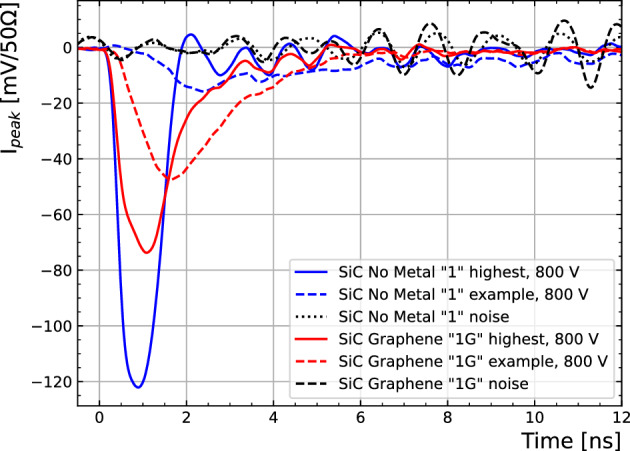
Corrected average over 1000 waveforms of Sample-1 and Sample-1G at $-800$ V at two different positions: Close to the edge and $\sim$ 500 $\upmu$m away. Black curves show the correction waveform to account for the laser system induced noise.

Due to pick-up noise caused by the laser system, the resulting average waveform has a coherent noise contribution. To correct for this effect, a measurement is done outside the active region of each sample which is used to subtract that contribution after baseline removal (see Fig. 4).

## Results

### Charge collection with radioactive source

**Figure 5:**
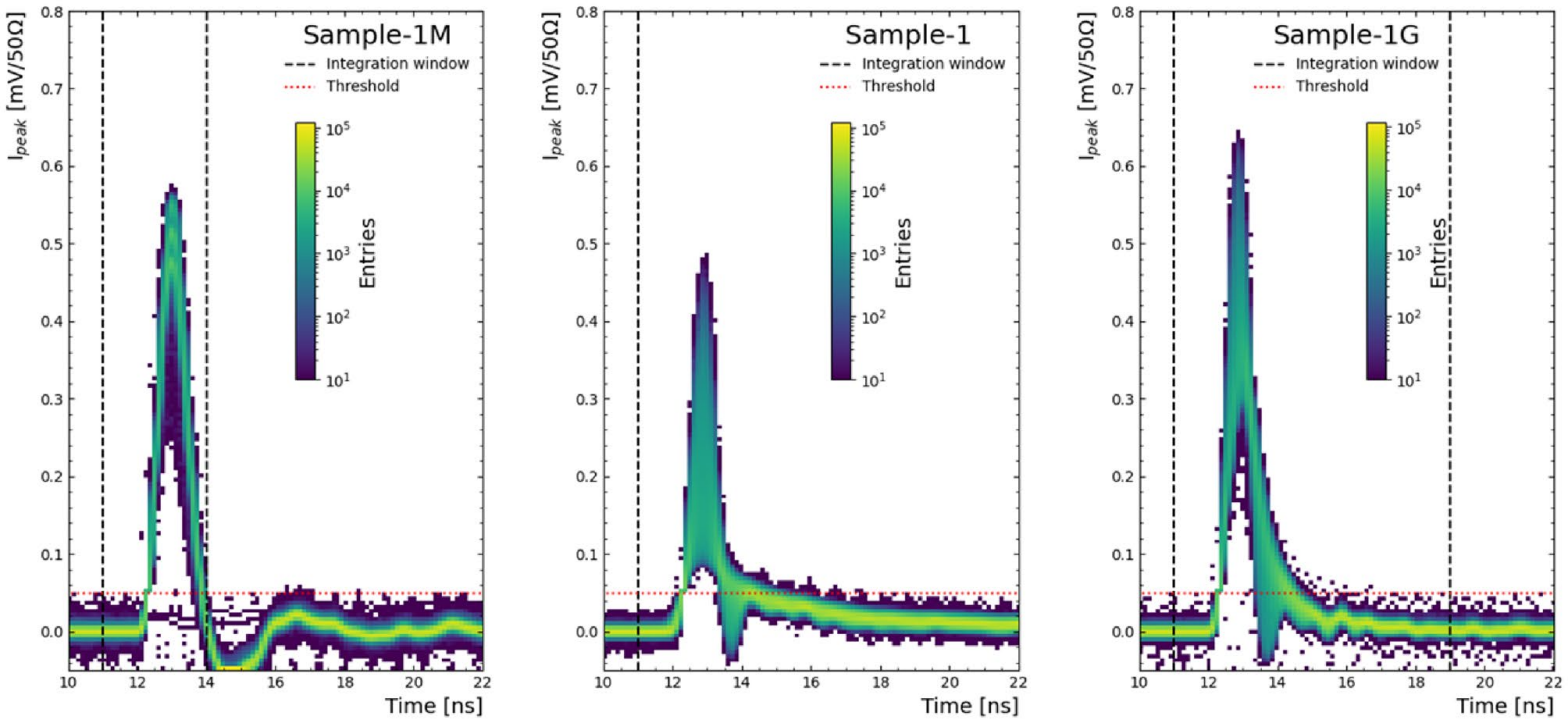
Transient signals obtained with the triple-alpha radioactive source for Sample-1M (left), -1 (centre) and -1G (right) under a bias voltage of $-500$ V. The black dashed lines show integration window while the red dotted line represents the threshold used during the data taking. Note that the integration window used for Sample-1 (28 ns) is longer than the plot range and thus not both ends are pictured.

The transient signals obtained under the triple-alpha radioactive source set-up are shown in Fig. 5. The waveforms obtained from all samples showcase a dip after the main transient pulse attributed to an impedance mismatch, causing a reflection. The integration window in Sample-1M was chosen before this undershoot, since the pulses from charge collection are faster. However, as the signals are longer in the other samples, the integration window for the charge determination is chosen longer, which in turn results in an under-estimation of the charge collection.

**Figure 6:**
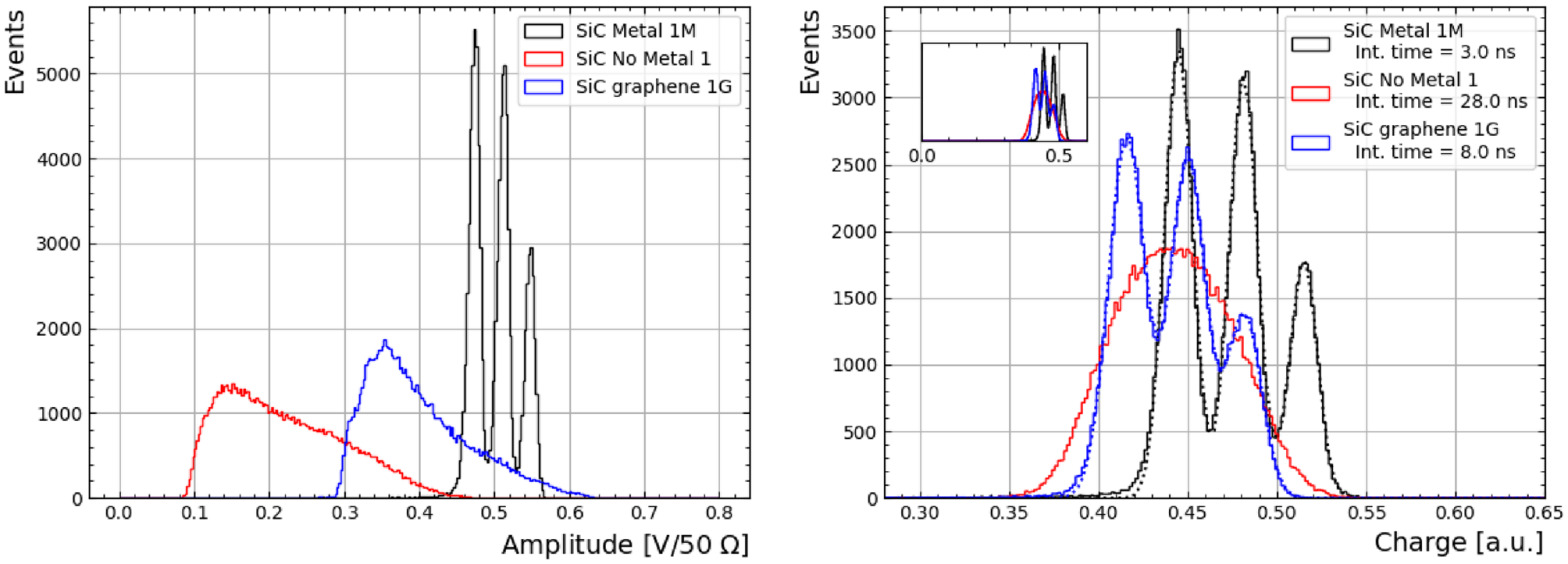
Amplitude (left) and charge distribution (right) obtained with the triple-alpha radioactive source for the Sample-1M (black, metallised), -1 (red, no metal) and -1G (blue, graphene) under a bias voltage of $-500$ V. The dashed lines show the result of a triple Gaussian distribution fit. The inset in the charge distribution shows the full range of charge amplitudes. Notice that different integration times were used across devices (see text).

Amplitude distribution for all samples under a bias voltage of -500 V are shown in Fig. 6 (left). Notably, Sample-1 has an overall lower amplitude distribution, although Sample-1M is the only detector showcasing the three peaks expected from the three alpha particle energies from the radioactive source. However - after integration - the same features appear in Sample-1G, as shown in Fig. 6 (right). As it will be observed in the following sections in more detail, the apparent charge collection loss in Sample-1 is a result of the higher resistance observed by the transient signal before reaching the bias ring with respect to the other samples, which reduces the waveform amplitudes away from it, to the point where noise is a large contribution in the integration. A 30 ns extra integration window was used in Sample-1 in order to recover charge levels comparable to Samples-1G and -1M. The same damping effect is seen in Sample-1G to a lesser degree.

A triple Gaussian distribution was fitted in the charge distributions of Samples-1G and -1M, the results of which are reported in Table 1, and shown in Fig. 6 (right) as a red dashed line. The peaks in Sample-1M are 7% higher with respect to Sample-1G, which is attributed to the integration window difference, as discussed in the previous paragraph. Note that this set-up is not optimised for spectroscopy, however, the spread of the peaks are found to be comparable in size, showing that the energy resolution in Sample-1G is recovered after signal integration.

It is also important to remark that while charge collection level is similar across all devices under test, they require different integration windows. This is a demerit for the geometry without conductive surface (i.e. Sample-1), as longer integration times are required to recover full charge collection, which makes it therefore more sensitive to noise.

**Table 1:** Results of the triple Gaussian fit in Fig. 6 (right) from exposing the samples with a triple-alpha source, with the corresponding energies according to the source spectrum.

	Source E_kin_					
	5.245 MeV		5.486 MeV		5.902 MeV	
	Q_1_	$\sigma _1$	Q_2_	$\sigma _2$	Q_3_	$\sigma _3$
1M	445.0 ± 0.1	7.9 ± 0.1	481.3 ± 0.1	7.8 ± 0.1	515.2 ± 0.1	7.6 ± 0.1
1G	415.9 ± 0.1	10.3 ± 0.1	450.2 ± 0.1	9.7 ± 0.1	481.7 ± 0.2	9.6 ± 0.2

Charge distribution obtained with Sample-1 was not adjusted as it did not showcase the three energy peak distribution (see text). All charge (Q_i_) and width ($\sigma _i$) units are in mV ns/50 $\Omega$, and uncertainties are statistical only.

### Position resolved study with TCT

**Figure 7:**
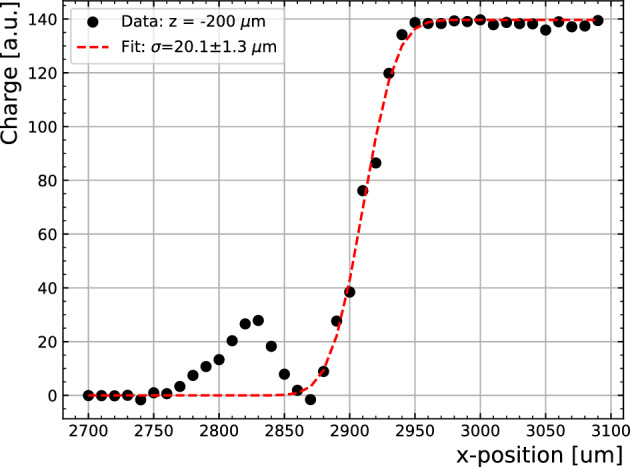
Charge collection across Sample-1G edge biased at $-300$ V with 10 $\upmu$m steps. After an error function fit, the focus size is measured to be 20±1 $\upmu$m. The peak observed at $x=(2760,2850)~\upmu$m corresponds to an open area away from the metallic collection ring and was not used for the fit.

The samples were installed in the Transient Current Technique set-up as described in the Methods section. The focused beam size onto the samples after optics has been measured to be about 20±1 $\mu$m, which is measured by an S-curve fit of the charge collected across the edge of a sample (see Fig. 7). Thus, hereafter positions are probed in 50 $\upmu$m steps.

**Figure 8:**
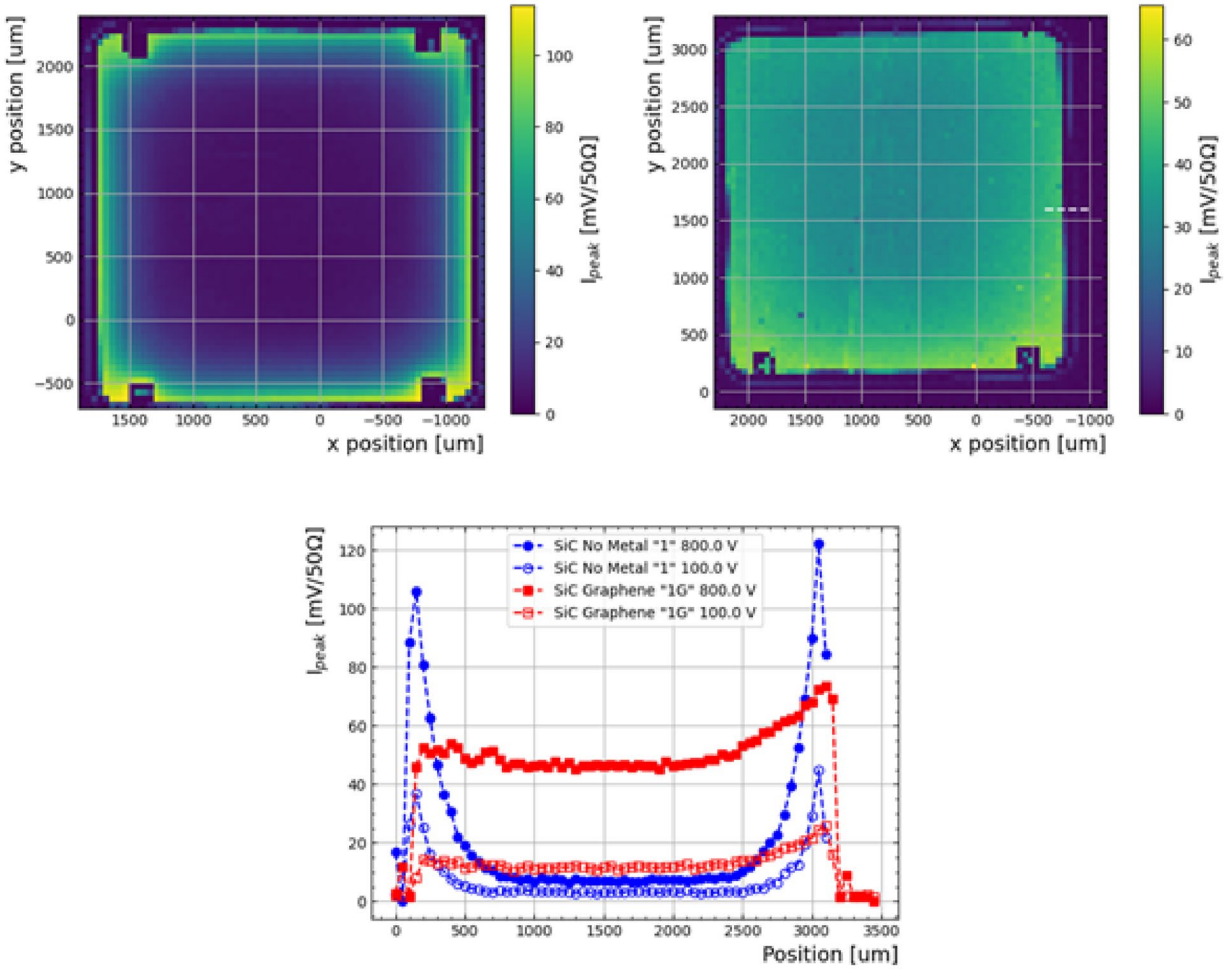
2D map of transient amplitudes at a $-300$ V bias voltage as a function of position of Sample-1 (top left) and Sample-1G (top right), the later with a white line denting the region where the data from Fig. 7 was taken (with a change of coordinate systems between measurements). Measurements at $-100$ and $-800$ V along the y-axis for either sample are shown (bottom).

The amplitude of the waveform as a function of position for both samples at $-300$ V bias voltage are shown in Fig. 8 (top). Here, the first effect of the graphene layer is observed: While for both samples the waveform amplitudes are reduced as a function of distance from the collection metal, this effect is much more abrupt on Sample-1. This is also observed in Fig. 8 (bottom), which shows the amplitude as a function of position along one axis. Interestingly, for Sample-1G only the edge closer to the wirebond shows a larger amplitude. Notice that the bias ring and the p+ implant in Sample-1G are interfaced with graphene, which has a contact resistivity larger than that of the metal-implant interface^23^. This demonstrates that the amplitude is damped away from the collection metal due to the resistive nature of the surface material. Since the resistivity of graphene is lower than that of the doped Silicon Carbide, the effect is not as pronounced in Sample-1G. This effect explains the amplitudes obtained in the radioactive source experiment not representing the triple peak expected for the radioactive source in Sample-1 and -1G.

**Figure 9:**
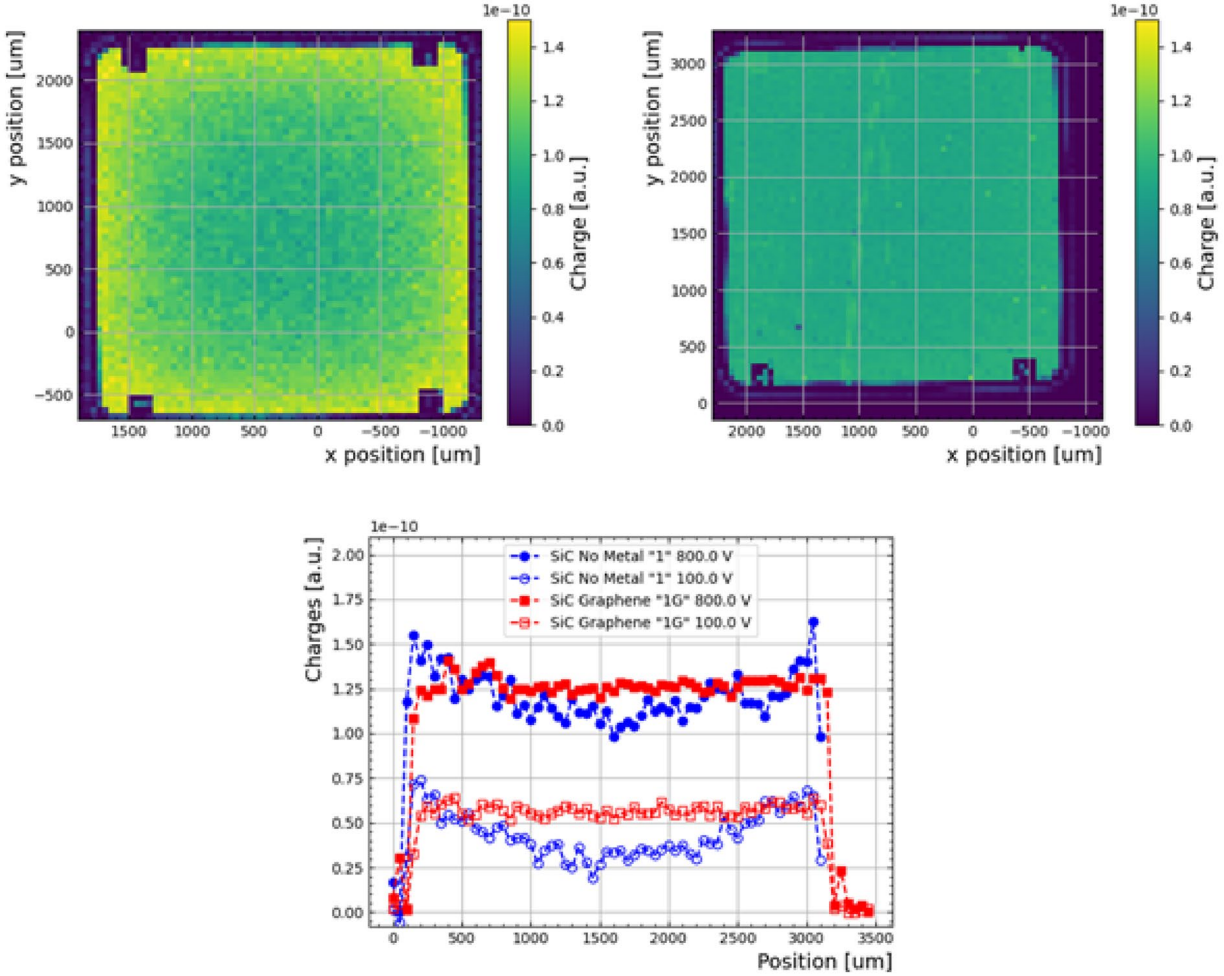
2D map of charges at a $-300$ V bias voltage as a function of position of Sample-1 (top-left) and Sample-1G (top-right). Measurements at $-100$ and $-800$ V along the y-axis for either sample are shown in the bottom plot.

Integrating the waveforms over time, the charge collection is calculated. In Fig. 9 (top) the charge collection as a function of position can be seen for either sample. As opposed to the amplitude, the integral of the waveform shows more uniformity. It is important to notice that the collection time drastically changes across samples at higher distance from the collection region, in particular for Sample-1. The charge collection across one direction at $-800$ V and $-100$ V is presented in Fig. 9 (bottom), which shows that the position dependence from the wirebond is not as apparent in terms of charge, most notably in Sample-1G. Again, this is compatible with the observation that the triple-alpha spectrum is recovered after integration for that sample, as seen in Fig. 6 which is not observed in Sample-1 possibly due to a low signal-to-noise ratio far from the bias ring, where the waveform amplitude is smaller.

**Figure 10:**
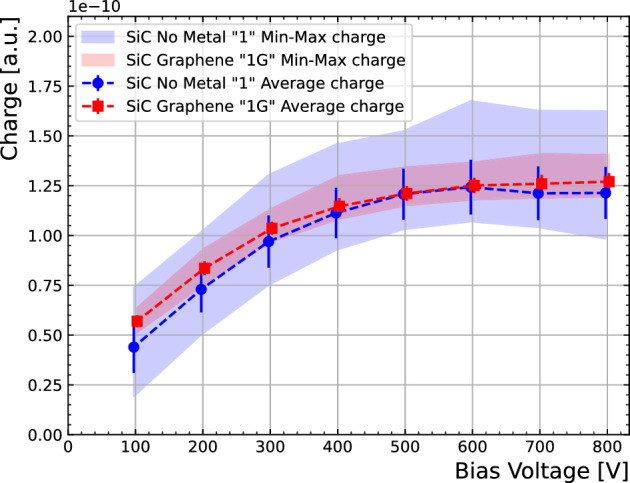
Average charge collection as a function of voltage. Coloured bands correspond to maximum and minimum values of charge.

To characterise charge collection as a function of voltage, the average charge is calculated and maximum and minimum charges are plotted as coloured bands in Fig. 10, extracted from the one-dimensional scan in Fig. 9(bottom). The saturation or depletion voltage is about -400 V for both samples thus the presence of graphene does not significantly affect the depletion in the bulk. This is compatible with CV measurements at wafer level reported in^23^.

## Discussion

Silicon carbide diodes with epitaxially grown graphene contacts have been produced at the IMB-CNM. To characterise the effect of graphene, the transient characteristics of such devices have been compared against samples without a graphene contact—with and without metal—from the same production wafer, via radioactive source exposure and transient current technique with a UV laser.

Graphene has shown a good charge collection behaviour, with a noticeable transient amplitude damping effect due to the resistivity of the surface material, although not as big as the reference sample without metal, making it a faster sensor than its other no-metal alternative. This effect could be useful for position-sensitive detection, by adding four connections to independent channels and using the difference in pulse height as a way to measure the particle position, similar to applications of the same principle in other solid state detectors^24,25^, while keeping a fast collection time.

However, uniform charge collection has been observed across the active region of the device, which is also observed in the form of recovered charge distribution when exposed to a triple-alpha radioactive source. Under the later conditions, the graphene sample shows only a slightly lower charge collection with respect to a sample with metallic contacts across its surface, owing to the need of longer integration window due its longer signals. This effect could be reduced with an encapsulation and readout electronics optimised for lower noise. This demonstrates that such design could be an alternative in applications where low energy ions or light (e.g UV light) need to be detected. No significant effect has been observed on the depletion and saturation voltages due to the presence of graphene.

Although future irradiation campaigns are needed, and are being scheduled to assess the behaviour of the prototypes after exposure in radiation harsh environments, these results show a promising behaviour of epitaxial graphene contacts in SiC detectors, with potential applications in low penetrating particle detectors such as soft X-rays, low energy ions, UV light detection and medical dosimetry where the presence of metal can encumber the radiation measurements.

## Data Availability

The data that support the findings of this study available from the corresponding author on reasonable request.
